# A Scallop Nitric Oxide Synthase (NOS) with Structure Similar to Neuronal NOS and Its Involvement in the Immune Defense

**DOI:** 10.1371/journal.pone.0069158

**Published:** 2013-07-26

**Authors:** Qiufen Jiang, Zhi Zhou, Leilei Wang, Lingling Wang, Feng Yue, Jingjing Wang, Linsheng Song

**Affiliations:** 1 Key Laboratory of Experimental Marine Biology, Institute of Oceanology, Chinese Academy of Sciences, Qingdao, China; 2 University of Chinese Academy of Sciences, Beijing, China; NIAID, United States of America

## Abstract

**Background:**

Nitric oxide synthase (NOS) is responsible for synthesizing nitric oxide (NO) from L-arginine, and involved in multiple physiological functions. However, its immunological role in mollusc was seldom reported.

**Methodology:**

In the present study, an NOS (CfNOS) gene was identified from the scallop *Chlamys farreri* encoding a polypeptide of 1486 amino acids. Its amino acid sequence shared 50.0~54.7, 40.7~47.0 and 42.5~44.5% similarities with vertebrate neuronal (n), endothelial (e) and inducible (i) NOSs, respectively. CfNOS contained PDZ, oxygenase and reductase domains, which resembled those in nNOS. The CfNOS mRNA transcripts expressed in all embryos and larvae after the 2-cell embryo stage, and were detectable in all tested tissues with the highest level in the gonad, and with the immune tissues hepatopancreas and haemocytes included. Moreover, the immunoreactive area of CfNOS distributed over the haemocyte cytoplasm and cell membrane. After LPS, β-glucan and PGN stimulation, the expression level of CfNOS mRNA in haemocytes increased significantly at 3 h (4.0-, 4.8- and 2.7-fold, respectively, *P* < 0.01), and reached the peak at 12 h (15.3- and 27.6-fold for LPS and β-glucan respectively, *P* < 0.01) and 24 h (17.3-fold for PGN, *P* < 0.01). In addition, TNF-α also induced the expression of CfNOS, which started to increase at 1 h (5.2-fold, *P* < 0.05) and peaked at 6 h (19.9-fold, *P* < 0.01). The catalytic activity of the native CfNOS protein was 30.3 ± 0.3 U mgprot^-1^, and it decreased significantly after the addition of the selective inhibitors of nNOS and iNOS (26.9 ± 0.4 and 29.3 ± 0.1 U mgprot^-1^, respectively, *P* < 0.01).

**Conclusions:**

These results suggested that CfNOS, with identical structure with nNOS and similar enzymatic characteristics to nNOS and iNOS, played the immunological role of iNOS to be involved in the scallop immune defense against PAMPs and TNF-α.

## Introduction

Nitric oxide synthase (NOS) is the enzyme that catalyzes the reaction from L-arginine to nitric oxide (NO), which is a ubiquitous and versatile gaseous signaling molecule, in the presence of five cofactors including nicotine adenine dinucleotide phosphate (NADPH), flavin adenine dinucleotide (FAD), flavin mononucleotide (FMN), tetrahydrobiopterin (BH_4_) and heme [1]. The active NOS is a homodimer, and each monomer contains two principal domains, an oxygenase domain at its N-terminus and a reductase domain at its C-terminus [2]. The oxygenase domain with Heme and BH_4_ binding sites is responsible for dimmer formation, while the reductase domain is able to bind FMN, FAD and NADPH [3].

According to the structure and activity features, vertebrate NOSs are divided into three isoforms, neuronal (n) NOS or type I, inducible (i) NOS or type II and endothelial (e) NOS or type III. Both nNOS and eNOS contain an autoinhibitory loop within the FMN binding region [4,5], whereas only nNOS possesses a PDZ (PSD-95 discs large/ZO-1 homology domain) domain at the N-terminal [6–8], while there exists neither autoinhibitory loop nor PDZ domain in iNOS. Besides, nNOS and eNOS depend on Ca^2+^ to produce constitutive NO at low levels (nM range), while iNOS can be induced by stress stimulation to synthesize NO at a higher level (μM range) independently of Ca^2+^. Furthermore, these three NOS isoforms are distributed in different tissues and involved in corresponding physiological activities. For example, nNOS is mainly expressed in neurons [7], and implicated in the regulation of neuronal activities by modulating the current flow (mainly Ca^2+^ flow) [9]. Released from vascular endothelial cells, eNOS is involved in the control of vascular tone, insulin secretion, airway tone, and the regulation of cardiac function and angiogenesis [10,11]. Another NO synthetase, iNOS, participates in the chronic neurodegeneration and immunologic diseases, including tumors, infectious and autoimmunity diseases [12,13].

In invertebrate, there hasn’t been any declaration upon the three-isoform constitution of NOS family. For example, there was only one NOS gene in *Drosophila* genome [14], which could be transcribed into multiple alternative RNA splicing variants [15], and a single copy of NOS ortholog was characterized from crustaceans [16–20]. In mollusc, NOSs were identified in the gastropods *Stramonita* *haemastoma* , *Lymnaea stagnalis*, *Aplysia* *californica* and *Limax valentianus*, the cephalopod *Sepia* *officinalis* , and the bivalve *Crassostrea* *gigas* and *Crassostrea* *virginica* [21–26], and all the identified NOSs belonged to one isoform which had higher similarities with nNOS than eNOS or iNOS from vertebrates. Though the composition of NOS family and the differentiation of NOS members in invertebrates were different from those in vertebrates, invertebrate NOSs had resembled broad involvements in various physiological activities. Arthropod NOSs could impact the tumor growth and the development [27,28], while mollusc NOSs were reported to participate in the synaptic transmission, learning and memory, as well as feeding [29–31]. Arthropod NOSs were also involved in the innate immunity [32,33], including the responses against the stimulations of *Vibrio* *penaeicida* , Poly I:C and LPS [18–20]. However, none of the identified mollusc NOSs have been claimed to be concerned with immune defense, and their roles in immune defense were far from well understood.

The Zhikong scallop *Chlamys farreri* is one of the most important marine economic species and contributes greatly to the aquaculture industry of China. In recent years, the scallops have suffered from severe diseases and mortalities, resulting in grievous loss to the aquaculture industry. Investigations of NOS functions on immune response would contribute to the further understanding of the immune defense mechanism in scallop and hopefully provide useful information to develop strategy for diseases control. The purposes of this study were (1) to clone the full-length cDNA of NOS from *C* *. farreri* (designated as CfNOS) and characterize its structure (2), to observe its mRNA distribution during ontogenesis and in different tissues, and its localization in scallop haemocytes (3), to examine the biochemical activity of native CfNOS protein, and (4) to investigate the response of the CfNOS transcripts to PAMPs (LPS, PGN and glucan) and TNF-α stimulation.

## Materials and Methods

### Ethics statement

The scallops used in the present study are marine cultured animals, and were collected from a local farm in Qingdao, Shandong Province, China, and maintained in the aerated seawater at 15-18 °C for a maximum of two weeks before processing. No specific permits are required for the described field studies, since the scallops in the local farm are provided for the local market-sellings. And the scallop *C* *. farreri* is not endangered or protected species.

Female Wistar rats were from Qingdao institute for the control of drug products (Qingdao, China), and the animal experiments were approved by the Animal Care and Use Committee at Qingdao institute for the control of drug products with a permit number of SCXX (Shandong) 20090007, which complied with the National Institute of Health Guide for the Care and Use of Laboratory.

All the experiments were conducted according to the regulations of local and central government.

### Tissue, embryos and larvae collection

Six tissues including hepatopancreas, kidney, adductor muscle, gonad, gill and mantle from six healthy adult scallops were collected as parallel samples. Haemolymph from these six scallops was also collected and then immediately centrifuged at 800×g, 4 °C for 10 min to harvest the haemocytes. All these samples were stored at -80 °C after addition of 1 mL Trizol reagent (Invitrogen, Carlsbad, CA) for subsequent RNA extraction.

Spawning of adult scallops was induced through thermal stimulation (4 °C above ambient temperature). The embryos or larvae at different stages were identified microscopically, including oocytes, fertilized eggs, 2-cell embryos, 4-cell embryos, 8-cell embryos, 16-cell embryos, 32-cell embryos, morula (6 hours post-fertilization, hpf), blastula (11 hpf), gastrula (18 hpf), trochophore (22 hpf), early D-hinged larvae (2 day post-fertilization, dpf), early veliger larvae (4 dpf), mid-veliger larvae (7 dpf) and late veliger larvae (23 dpf). Six duplicate samples were collected at each stage, resuspended in 1 mL Trizol reagent, and then stored in liquid nitrogen immediately for subsequent RNA extraction.

### PAMPs and TNF-α stimulation

One hundred and thirty-eight adult scallops were employed for the PAMPs (LPS, PGN and glucan) stimulation experiment. One hundred and twenty scallops were randomly divided into 4 groups and each group contained 30 individuals. The scallops receiving an injection of 50 µL of phosphate buffered saline (PBS, 136.89 mmol L^-1^ NaCl, 2.68 mmol L^-1^ KCl, 8.10 mmol L^-1^ Na_2_HPO_4_, 1.47 mmol L^-1^ KH_2_PO_4_, pH 7.4) were employed as control group. And those receiving an injection of 50 µL of LPS from *Escherichia coli* 0111:B4 (Sigmae Aldrich, 0.5 mg mL^-1^ in PBS), PGN from *Staphylococcus aureus* (Sigmae Aldrich, 0.8 mg mL^-1^ in PBS), and β-glucan from *Saccharomyces cerevisiae* (Sigmae Aldrich, 1.0 mg mL^-1^ in PBS) were employed as LPS, PGN and β-glucan treated groups, respectively. After treatment, the scallops were returned to water tanks and 6 individuals were randomly sampled at 3, 6, 12, 24 and 48 h post-injection. Six individuals were randomly sampled at 0 h from the rest 18 scallops as the blank group. The haemolymph was collected, and centrifuged at 800×g, 4 °C for 10 min to harvest the haemocytes for RNA preparation.

Another sixty scallops were employed for the TNF-α stimulation experiment. Forty scallops were randomly divided into 2 groups. The scallops in the control and stimulation groups received an injection of 50 µL of PBS and 50.0 ng mL^-1^ human TNF-α (Invitrogen, in PBS), respectively. These treated scallops were returned to water tanks, and six individuals were randomly sampled at 1, 3, 6 and 9 h post-injection from the two groups. Six individuals were randomly sampled at 0 h in the rest 12 scallops employed as the blank group. The haemocytes were harvested and stored as described above.

### RNA isolation and cDNA synthesis

Total RNA was isolated from the embryos, larvae, tissues, and the tested haemocytes using Trizol reagent according to the protocol. The first-strand synthesis was carried out based on Promega M-MLV RT Usage information using the DNase I (Promega)-treated total RNA as template and oligo(dT)-adaptor as primer (Table 1). The reaction was performed at 42 °C for 1 h, terminated by heating at 95 °C for 5 min. The cDNA mix was diluted to 1:100 and stored at -80 °C for subsequent SYBR Green fluorescent quantitative real-time PCR.

**Table 1:** Sequences of the primers used in the experiment.

Primer	Sequence (5’–3’)	Sequence information
P1 (forward)	AGTTTCCAGTCTCAAGGCGTTAC	Real-time CfNOS primer
P2 (reverse)	CGCGGTTCTTCTGTTCATTCT	Real-time CfNOS primer
P3 (forward)	CAAACAGCAGCCTCCTCGTCAT	Real-time actin primer
P4 (reverse)	CTGGGCACCTGAACCTTTCGTT	Real-time actin primer
P5 (forward)	ATCCTTCCTCCATCTCGTCCT	CfEF-1a specific primer
P6 (reverse)	GGCACAGTTCCAATACCTCCA	CfEF-1a specific primer
F631 (forward)	TGTTGGACTGTGGNGGNCTGGAATT	Homologous cloning primer
R735 (reverse)	GGAGGNACDATCCACACCCAGTC	Homologous cloning primer
3F1 (forward)	TTGGTGCCAGGGACTTCT	CfNOS specific primer
3F2 (forward)	AGTTTCCAGTCTCAAGGCGTTAC	CfNOS specific primer
3F3 (forward)	GGCTGTGAAGTTCTCCGCTAAA	CfNOS specific primer
3F4 (forward)	TGGGATACTCTCCCGCCTGCATAATG	CfNOS specific primer
3F5 (forward)	ACACTCAACCGAACCTGGTGGAGATTC	CfNOS specific primer
3F6 (forward)	CCTCTACTTTGGCTGTAGGCAGAATGATG	CfNOS specific primer
3F7 (forward)	GTAACTCAAAACGGAATCGGAACTT	CfNOS specific primer
5R1 (reverse)	GACTTGGTCTTGTCCCTGTCTTT	CfNOS specific primer
5R2 (reverse)	ACGCCTTGAGACTGGAAACTATG	CfNOS specific primer
5R3 (reverse)	CCCATCTTGGTCGCAATAAC	CfNOS specific primer
5R4 (reverse)	AAATGGTGCCGCAGTAAAT	CfNOS specific primer
5R5 (reverse)	CCATCGGTTCGTGCCGGAAAAATAGTA	CfNOS specific primer
5R6 (reverse)	ACGAGGTTCTCCAACCGGTCGAGTG	CfNOS specific primer
5R7 (reverse)	CTGGGAGGATGGACTGTGTAAGTTGAT	CfNOS specific primer
5R8 (reverse)	GTTTTTACTCACATCCCCGTTCTCCTT	CfNOS specific primer
P7	GGCCACGCGTCGACTAGTACT_17_	Oligo(dT)-adaptor
P8	GGCCACGCGTCGACTAGTACG_10_	Oligo(dG)-adaptor
M13-47	CGCCAGGGTTTTCCCAGTCACGAC	pMD18-T simple vector primer
RV-M	GAGCGGATAACAATTTCACACAGG	pMD18-T simple vector primer
RbF (forward)	TGAGTCGACATATGGACGAAAAACAAGTATTCAGCGACAC	CfNOS recombination primer
RbR (reverse)	CAGGCGGCCGCAGATGTCTATTTCCTTGTCGTTAT	CfNOS recombination primer
T7pro	TAATACGACTCACTATAGGG	pEASY-E1 vector primer
T7ter	GCTAGTTATTGCTCAGCGG	pEASY-E1 vector primer

### The homologous analysis and cloning of the full-length CfNOS cDNA

A couple of degenerate primers (Table 1) were designed based on the nucleotide sequence of *Thais* *Haemastoma* NOS (FR667655.3), *L* *. stagnalis* NOS (AAC17487.1), *A* *. californica* NOS (AAK83069.1), *S* *. officinalis* NOSa (AAS93626.1), *S* *. officinalis* NOSb (AAS93627.1) and *C* *. gigas* NOS (EKC33784.1) (Table 2). A fragment of 334 bp was amplified by using the degenerate primers. The PCR products were gel-purified and cloned into the pMD18-T simple vector (Takara, Japan). After being transformed into the competent cells of *E. coli* Top10F’, the positive recombinants were identified through anti-ampicillin selection and PCR screening with sense vector primer RV-M and antisense vector primer M13-47 (Table 1). Three of the positive clones were sequenced on an ABI 3730 XL Automated Sequencer (Applied Biosystems). According to the obtained sequence, specific primers (Table 1) were designed for cloning of the full-length cDNA by rapid amplification of cDNA ends (RACE) approach. PCR amplification to clone the 3’ end of CfNOS was carried out using sense primer 3F1~7 and antisense primer Oligo(dT)-adaptor P7, while sense primer Oligo(dG)-adaptor P8 and antisense primer 5R1~8 were used to get the 5’ end according to the Usage Information of 5’ RACE system (Invitrogen). The obtained PCR products were purified and sequenced as described above. The sequences were overlapped, verified, and subjected to cluster analysis.

**Table 2:** Related information of nitric oxide synthase (NOS) used in the homologous analysis, multiple sequences alignment and phylogenetic analysis.

Species	Code name	Protein	Accession number	Similarity	Identity
*Homo sapiens*	Human	nNOS	AAA62405.1	53.6%	43.2%
*Homo sapiens*	Human	iNOS	NP_000616.3	44.2%	36.1%
*Homo sapiens*	Human	eNOS	NP_000594.2	45.7%	36.1%
*Rattus norvegicus*	Rat	nNOS	AAC52782.1	52.8%	42.4%
*Rattus norvegicus*	Rat	iNOS	AAC13747.1	43.5%	35.1%
*Rattus norvegicus*	Rat	eNOS	NP_068610.1	45.7%	35.9%
*Bos taurus*	Bovine	nNOS	XP_002694631.2	53.5%	42.6%
*Bos taurus*	Bovine	iNOS	NP_001070267.1	44.3%	35.6%
*Bos taurus*	Bovine	eNOS	NP_851380.2	46%	36.3%
*Gallus gallus*	Chicken	nNOS	XP_425296.2	54.3%	43.8%
*Gallus gallus*	Chicken	iNOS	NP_990292.1	43.4%	34.3%
*Gallus gallus*	Chicken	eNOS	AFD20677.1	40.7%	33.7%
*Xenopus* (Silurana)*tropicalis*	*Xenopus*	nNOS	XP_002938130.1	54.7%	43.2%
*Xenopus* (Silurana)*tropicalis*	*Xenopus*	iNOS	XP_002935342.1	44%	35.3%
*Xenopus* (Silurana)*tropicalis*	*Xenopus*	eNOS	ACV74251.1	47%	37.3%
*Xenopus laevis*	*X laevis*	nNOS	NP_001079155.1	54.5%	43.1%
*Danio rerio*	Danio	nNOS	NP_571735.1	53.3%	43.6%
*Danio rerio*	Danio	iNOSa	NP_571735.1	44.5%	35.1%
*Danio rerio*	Danio	iNOSb	NP_001098407.1	42.5%	33.6%
*Branchiostoma* *floridae*	Branchiostoma	NOS	AAQ02989.1	53.4%	41.1%
*Ciona* *intestinalis*	Ascidean	nNOS	XP_002120267.1	50%	37.5%
*Drosophila melanogaster*	Drosophila	NOS	AAC46882.1	45.3%	35.6%
*Daphnia* *magna*	*Daphnia*	NOS1	ACQ55298.1	43.4%	33.2%
*Daphnia* *magna*	*Daphnia*	NOS2	ACQ55299.1	38.8%	29.1%
*Scyllaparamamosain*	Scylla	NOS	CCC18661.1	43.8%	35.9%
*Litopenaeusvannamei*	Litopenaeus	NOS	ADD63793.1	44.2%	36.3%
*Lehmannia* *valentiana*	*Lehmannia*	limNOS	BAF73722.1	57.5%	47.7%
*Lymnaea stagnalis*	Lymnaea	NOS	AAC17487.1	42.9%	34.6%
*Lymnaea stagnalis*	Lymnaea	NOS2	AAW88577.1	40.7%	32.6%
*Aplysia* *californica*	*Aplysia*	NOS	AAK83069.1	55.7%	45.6%
*Aplysia* *californica*	*Aplysia*	NOS2	AAK92211.3	44.6%	35.7%
*Sepia* *officinalis*	Sepia	NOSa	AAS93626.1	52.8%	44.3%
*Sepia* *officinalis*	Sepia	NOSb	AAS93627.1	52.5%	44.1%
*Crassostrea* *gigas*	*Crassostrea*	NOS	EKC33784.1	68.9%	59.7%
*Thais* *haemastoma*	Thais	NOS1	CBV37021.3	66.2%	55.1%
*Chlamys farreri*	Scallop	CfNOS	KC237281	100%	100%
*Strongylocentrotus purpuratus*	Urchin	NOS1	XP_003729305.1	46.9%	36.6%

### Sequence analysis

The homology searches of the cDNA sequence and amino acid sequence of CfNOS were conducted with BLAST algorithm at the National Center for Biotechnology Information (http://www.ncbi.nlm.gov/blast). The deduced amino acid sequence was analyzed with the Expert Protein Analysis System (http://www.expasy.org). The presence and location of signal peptide was predicted using the SignalP 3.0 program (http://www.cbs.dtu.dk/services/SignalP). The protein domains were predicted with the simple modular architecture research tool (SMART) version 7.0 (http://www.smart.emblheidelberg.de/). The multiple sequence alignment of CfNOS with other NOSs was performed with the ClustalW multiple alignment program (http://www.ebi.ac.uk/clustalw/) and displayed through the multiple alignment show program (http://www.bio-soft.net/sms/index.html). An unrooted phylogenic tree was constructed based on the deduced amino acid sequence of CfNOS and other NOSs by the Maximum Likelihood (ML) algorithm using the Seaview 4.0 software [34,35].

### Real-time PCR analysis of CfNOS mRNA expression

The quantitative real-time PCR amplification was carried out in a total volume of 25.0 µL, containing 2.0 µL 100 × diluted cDNA, 12.5 µL 2 × SYBR Premix Ex Taq^TM^ II (Applied Biosystems, USA), 0.5 µL of each primers (10 mmol L^-1^) and 9.5 µL DEPC-water. For CfNOS, a 91 bp product was amplified with the primers P1 and P2 (Table 1). Two β-actin primers and two CfEF-1α (elongation factor 1 alpha from scallop *C* *. farreri* ) primers (Table 1) were used to amplify 94 and 86 bp fragments, respectively, as internal controls to verify the successful reverse transcription and calibrate the cDNA template. The β-actin was referenced in the tissue distribution analysis and stimulation experiments, while the CfEF-1α was used in the developmental expression analysis of CfNOS mRNA as described by Shi et al. [36]. The SYBR Green real-time PCR assay was carried out in an ABI PRISM 7300 Sequence Detection System (Applied Biosystems) according to the manual. After the PCR program, dissociation curve analysis of amplification products was performed to confirm that only one PCR product was amplified and detected. To maintain consistency, the baseline was set automatically by the ABI 7300 SDS software V 2.0. The 2^−ΔΔCT^ method was used to analyze the relative expression level of CfNOS [37].

### Recombinant expression of CfNOS protein

The cDNA fragment encoding the oxygenase domain and FMN binding region of CfNOS was amplified from the cDNA library of scallops using Promega Taq polymerase with specific primers RbF and RbR (Table 1). The PCR products were gel-purified and cloned into *pEASY*-E1 expression vector (Transgen, China) according to the Usage Information of *pEASY*-E1 expression system. The recombinant plasmid (*pEASY*-E1-CfNOS) was transformed into Trans1-T1 phage resistant chemically competent cell of *E. coli* (Transgen). The forward positive clones were screened by PCR with vector primer T7pro and antisense primer RbR (Table 1), and further confirmed by sequencing. The valid recombinant plasmid (*pEASY*-E1-CfNOS) was extracted and transformed into *E. coli* BL21 (DE3)-Transetta (Transgen).

Positive transformants were incubated in LB medium (containing 100 mg mL^-1^ ampicillin) at 37 °C with shaking at 220 rpm. When the culture mediums reached OD_600_ of 0.5-0.7, the cells were incubated for additional 4 hours with the induction of IPTG at the final concentration of 1.0 mmol L^-1^. The recombinant protein of CfNOS (designated rCfNOS) were purified by a Ni^2+^ chelating Sepharose column (GE Healthcare), pooled by elution with 400 mmol L^-1^ imidazole under denatured condition (8 mol L^-1^ urea). The purified protein was refolded in gradient urea-TBS glycerol buffer (50 mmol L^-1^ Tris-HCl, 50 mmol L^-1^ NaCl, 10% glycerol, 2 mmol L^-1^ reduced glutathione, 0.2 mmol L^-1^ oxide glutathione, a gradient urea concentration of 6, 4, 3, 2, 1, and 0 mol L^-1^ urea in each gradient, pH 7.4, each gradient at 4 °C for 12 h). Then the resultant proteins were separated by reducing 12% SDS-polyacrylamide gel electrophoresis (SDS-PAGE), and visualized with Coomassie bright blue R250. The concentration of purified rCfNOS was quantified by BCA method, and then the obtained protein was stored at -80 °C before use.

### Preparation of antibody and western blotting analysis

For preparation of antibody against CfNOS, the renatured rCfNOS was dialyzed continuously against ddH_2_O before it was freeze concentrated. And the protein rCfNOS was immuned to 6 weeks old rats to acquire polyclonal antibody.

After SDS-PAGE, the samples (purified rCfNOS) were electrophoretically transferred onto a 0.45 mm pore nitrocellulose membrane at 250 mA for 3 h. The membrane was blocked with PBS containing 5% skim milk powder at 37°C for 1 h. After three washings with PBS containing 0.05% Tween-20 (PBST), the membrane was incubated with rCfNOS antibody at 37°C for 1 h, and then washed three times with PBST. Antibody binding was detected with goat-anti-rat Ig-alkaline phosphatase conjugate (Southern Biotech) diluted 1:4000 in PBS at 37 °C for 1 h, and washed three times with PBST. Then protein band was stained with freshly prepared substrate solution (100 mmol L^-1^ NaCl, 100 mmol L^-1^ Tris and 5 mmol L^-1^ MgCl_2_, pH 9.5) containing nitroblue tetrazolium (NBT, Sigma) and 5-bromo-4-chloro-3-indolyphosphate (BCIP, Sigma), and the reaction was stopped by washing with distilled water. Rats’ non-immune serum was used as negative control.

### Immunofluorescence and immunocytochemistry (IF/ICC) assay

The sub-cellular localization of CfNOS protein in scallop haemocytes was observed by immunofluorescence and immunocytochemistry (IF/ICC) method using a laser confocal microscopy (Zeiss, Germany). Haemolymph was aspirated by a syringe from the adductor muscle of scallop in ALS (Alseve) buffer (20.8 g L^-1^ glucose, 8.0 g L^-1^ sodium citrate, 3.36 g L^-1^ ethylene diamine-tetraacetic acid and 22.5 g L^-1^ NaCl, pH 7.0 and 1000 mOsmol) with the ratio of 1:1. The suspension was centrifuged at 800×g for 10 min and the cells were resuspended in modified Leibovitz L-15 medium (supplemented with 20.2 g L^-1^ NaCl, 0.54 g L^-1^ KCl, 0.6 g L^-1^ CaCl_2_, 1.0 g L^-1^ MgSO_4_, 3.9 g L^-1^ MgCl_2_, 20.8 g L^-1^ glucose, 10% FCS, 100 µg mL^-1^ penicillin G, 100 µg mL^-1^ streptomycin, 40 µg mL^-1^ gentamicin and 0.1 µg mL^-1^ amphotericin B at pH 7.0 and 1000 mOsm). One milliliter of the adjusted haemocyte suspension (8 × 10^5^ cell mL^-1^) was added to the glass bottom dishes, which were then placed in the 18 °C incubator for 3 h. After three washings with L-15 medium, the haemocytes were prefixed with 1 mL of 0.0083% glutaraldehyde for 15 min and fixed with 1 mL of 4% paraformaldehyde in L-15 medium at room temperature for 20 min. Then, the cells were permeabilized with 0.5% Triton X-100 at room temperature for 10 min and blocked with 3% BSA in L-15 medium at 37 °C for 30 min. Subsequently, the haemocytes were incubated with rCfNOS antibody (1:1000) at 4 °C overnight, and then with fluorescein Alexa-488 conjugated goat-anti-rat IgG (1:1000) at 18 °C in dark for 1 h. After dyeing with DAPI (Beyotime biotechnology, China), the haemocytes were mounted with a drop of 50% glycerin and observed under the laser confocal microscopy.

### NOS activity assay

Native CfNOS protein was isolated from scallop haemocytes by immunoprecipitation and its catalytic activity was examined by NOS enzymatic method. The haemocytes were resuspended in cell lysis buffer (Beyotime biotechnology, China) and lysed by ultrasonic crusher for 5 min in ice-water bath. After the centrifugation at 8000×g, 4 °C for 30 min, the supernatant was collected and adjusted to the final protein concentration of 2 mg mL^-1^. Five hundred microliter supernatant was pre-incubated with equivalent volume of rCfNOS antibody (2 mg mL^-1^ of protein concentration) with slight agitation at 4 °C for 1 h. The solutions were loaded into the Protein G GraviTrap column (GE, USA), and the targeted proteins were eluted according to the manual. The obtained product was incubated with the elution buffer, Spermidine Trihydrochloride (Spd, selective inhibitor of nNOS, 0.01 mmol L^-1^, Beyotime biotechnology), NG-Nitro-L-arginine Methyl Ester Hydrochloride (L-NAME, selective inhibitor of eNOS, 0.01 mmol L^-1^, Beyotime biotechnology), and S-Methylisothiourea Sulfate (SMT, selective inhibitor of iNOS, 0.01 mmol L^-1^, Beyotime biotechnology) at 18 °C for 30 min, respectively. The absorbance of the mixture was measured with a microtiter plate reader (BioTek, USA) at 530 nm, and the activity of NOS was determined following the manual of total NOS detection kit (Jiancheng, Nanjing, China). Each experiment was repeated in triplicate, and the elution buffer was used as blank. Results were expressed as unit activity per milligram protein in the obtained elution (U mgprot^-1^).

### Statistical analysis

All data was given as means ± SD. The data was subjected to one-way analysis of variance (one-way ANOVA) followed by a multiple comparison (S–N–K). Differences were considered significant at *P* < 0.05.

## Results

### The molecular characters of CfNOS cDNA

A 5023 bp nucleotide sequence representing the complete cDNA sequence of CfNOS was obtained. The cDNA sequence consisted of a 5’ untranslated region (UTR) of 140 bp, a 3’ UTR of 422 bp with a poly(A) tail, and an open reading frame (ORF) of 4461 bp encoding a polypeptide of 1486 amino acids with a predicted molecular weight of 167.2 kDa and a theoretical isoelectric point of 7.9. The sequence was deposited in GenBank under accession no. **KC237281**. The deduced amino acid sequence of CfNOS had highest similarity with NOS from *C* *. gigas* (EKC33784.1, 68.9% similarity), and shared 50~54.7, 40.7~47.0 and 42.5~44.5% similarities with nNOS, eNOS and iNOS from chordates, respectively (Table 2).

**Figure 1:**
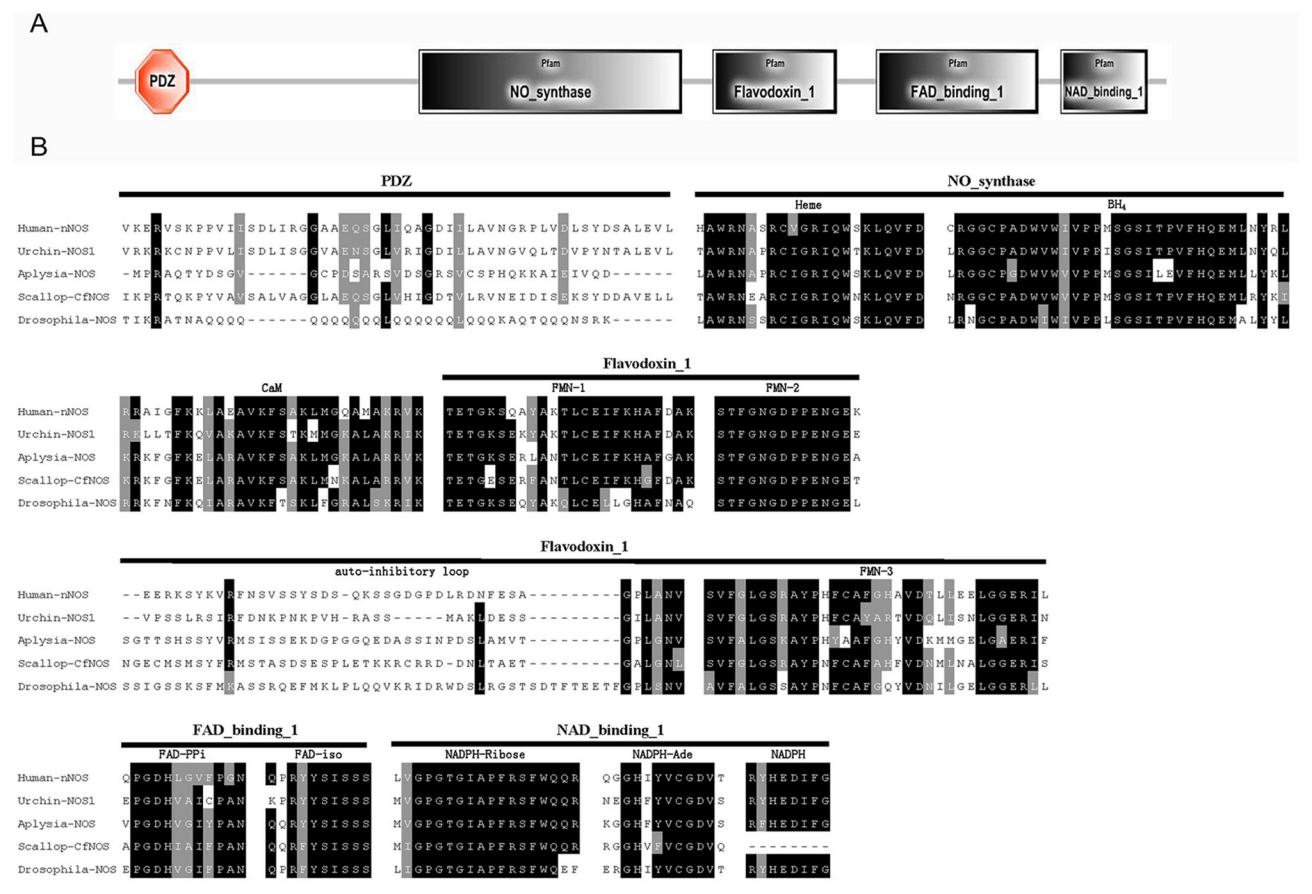
Multiple sequence alignment analysis of putative domains of CfNOS and other NOSs deposited in GenBank. (A) The solid straight line indicated the major domains or binding regions predicted by SMART program, including a PDZ domain (Asp25–Asp100), an NO synthase domain (Gly427–Lys799), a flavodoxin 1 region (Ile843-Trp1018), an FAD (Thr1075-Trp1304) and an NAD binding region (Met1336-Thr1459). (B) The species and the GenBank accession numbers were as follows: *Homo sapiens* (AAA62405.1), *Drosophila melanogaster* (AAC46882.1), *Aplysia* *californica* (AAK83069.1), *Chlamys farreri* and *Strongylocentrotus purpuratus* (XP_003729305.1). The black shadow region indicated positions where all sequences shared the same amino acid residues. Similar amino acids were shaded in grey. Gaps were indicated by hyphens to improve the alignment. The binding sites for heme (Thr488–Asp509), BH_4_ (Asn750–Ile782), Ca^2+^/calcium (CaM) (Lys812–Lys840), FMN (Thr847–Lys870, Ser893–Thr906, Ser968-Ser1000), FAD (Ala1118-Asn1129, Gln1261-Ser1270), NADPH (Met1336-Arg1353, Arg1441-Gln1452), and the location of an autoinhibitory loop (Asn922–Leu965) were identified by multiple sequences alignment.

No signal peptide was predicted by SignalP software analysis. Multiple sequence alignment revealed the domains of PDZ (Asp25–Asp100), oxygenase (Gly427–Lys799) and reductase (Ile843-Thr1459), and the latter two were connected by the binding site for CaM (Lys812–Lys840). SMART program analysis predicted a flavodoxin 1 region (Ile843-Trp1018), an FAD (Thr1075-Trp1304) and an NAD binding region (Met1336-Thr1459), which were included in the reductase domain (Figure 1A). Besides, important conserved sites were also identified from the alignment, including the binding sites for heme (Thr488–Asp509), BH_4_ (Asn750–Ile782), FMN (Thr847–Lys870, Ser893–Thr906, Ser968-Ser1000), FAD (Ala1118-Asn1129, Gln1261-Ser1270), NADPH (Met1336-Arg1353, Arg1441-Gln1452) and the location of an autoinhibitory loop (Asn922–Leu965) (Figure 1B).

**Figure 2:**
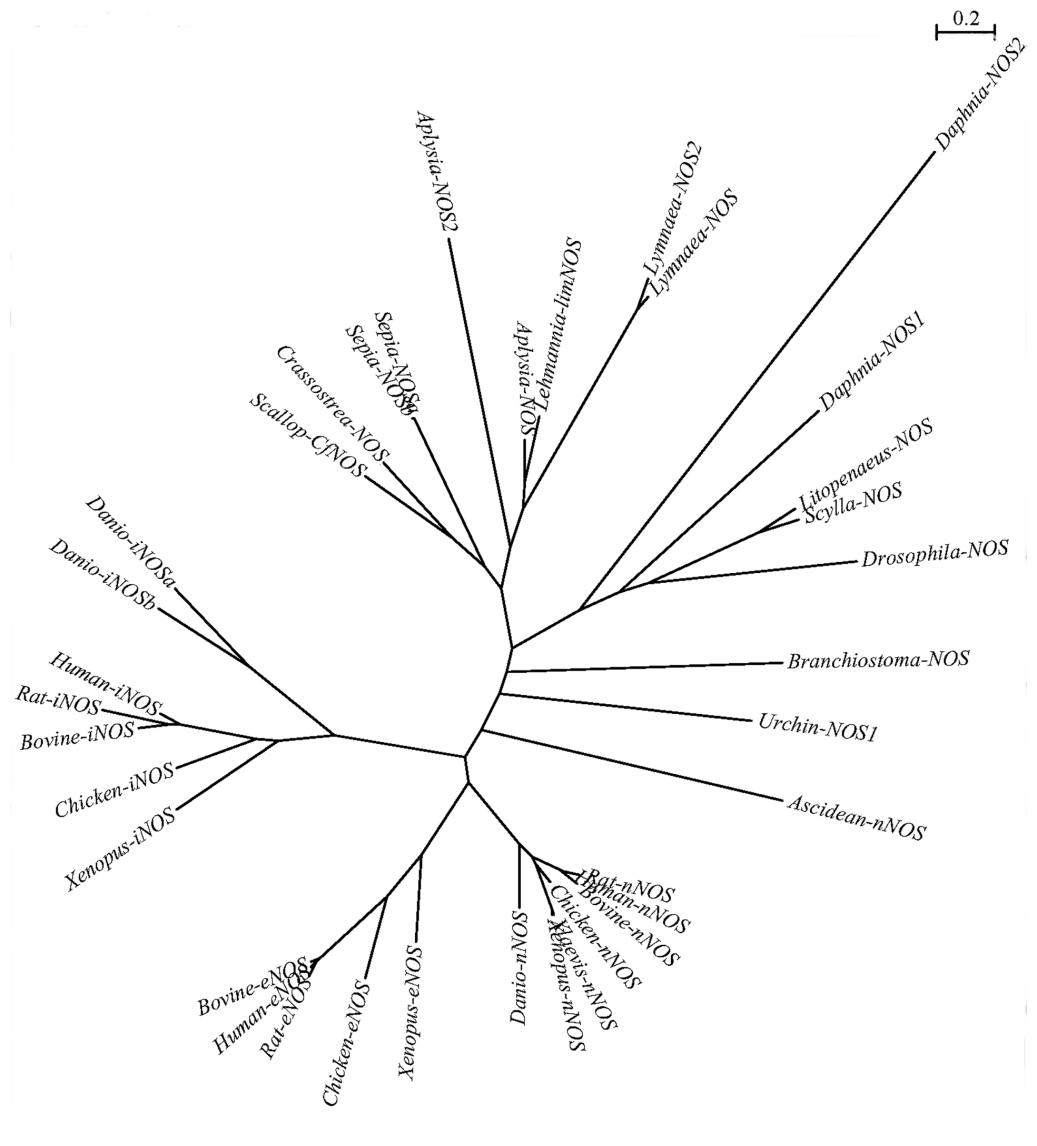
Maximum likelihood (ML) algorithm tree based on the amino acid sequences of NOSs. The scale bar represents conversion of branch length to genetic distance between clades (0.1 = 10% genetic distance). The protein sequences used for phylogenetic analysis include: nNOSs from *H. sapiens* (AAA62405.1), *Rattus norvegicus* (AAC52782.1), *Bos taurus* (XP_002694631.2), *Gallus gallus* (XP_425296.2), *Xenopus* (Silurana) *tropicalis* (XP_002938130.1), *Xenopus laevis* (NP_001079155.1), *Ciona* *intestinalis* (XP_002120267.1), iNOSs from *H. sapiens* (NP_000616.3), *R. norvegicus* (AAC13747.1), *B. Taurus* (NP_001070267.1), *G. gallus* (NP_990292.1), *X* *. tropicalis* (XP_002935342.1), *Danio rerio* (NP_571735.1 and NP_001098407.1), eNOSs from *H. sapiens* (NP_000594.2), *R. norvegicus* (NP_068610.1), *B. Taurus* (NP_851380.2), *G. gallus* (AFD20677.1), *X* *. tropicalis* (ACV74251.1), and NOSs from *Branchiostoma* *floridae* (AAQ02989.1), *D. melanogaster* (AAC46882.1), *Daphnia* *magna* (ACQ55298.1 and ACQ55299.1), *Scyllaparamamosain* (CCC18661.1), *Litopenaeusvannamei* (ADD63793.1 and BAF73722.1), *Lymnaea stagnalis* (AAC17487.1 and AAW88577.1), *A* *. californica* (AAK83069.1 and AAK92211.3), *Sepia* *officinalis* (AAS93626.1 and AAS93627.1), *Crassostrea* *gigas* (EKC33784.1), *S. purpuratus* (XP_003729305.1).

An unrooted phylogenetic tree was constructed using Maximum Likelihood (ML) based on the multiple sequence alignment of CfNOS and other NOSs (Figure 2). There were three distinct groups in the tree. CfNOS was clustered with the NOSs from gastropod, cephalopod, and other bivalve NOSs to form the mollusc group at the top of the phylogenetic tree. The NOSs from arthropods were gathered together to form the second group, while those from deuterostomes were clumped into the third group. In the vertebrate clade of the deuterostome group, the nNOSs and eNOSs were firstly bunched together, and formed a sister branch to iNOSs.

### Recombinant protein of CfNOS and its polyclonal antibody

**Figure 3:**
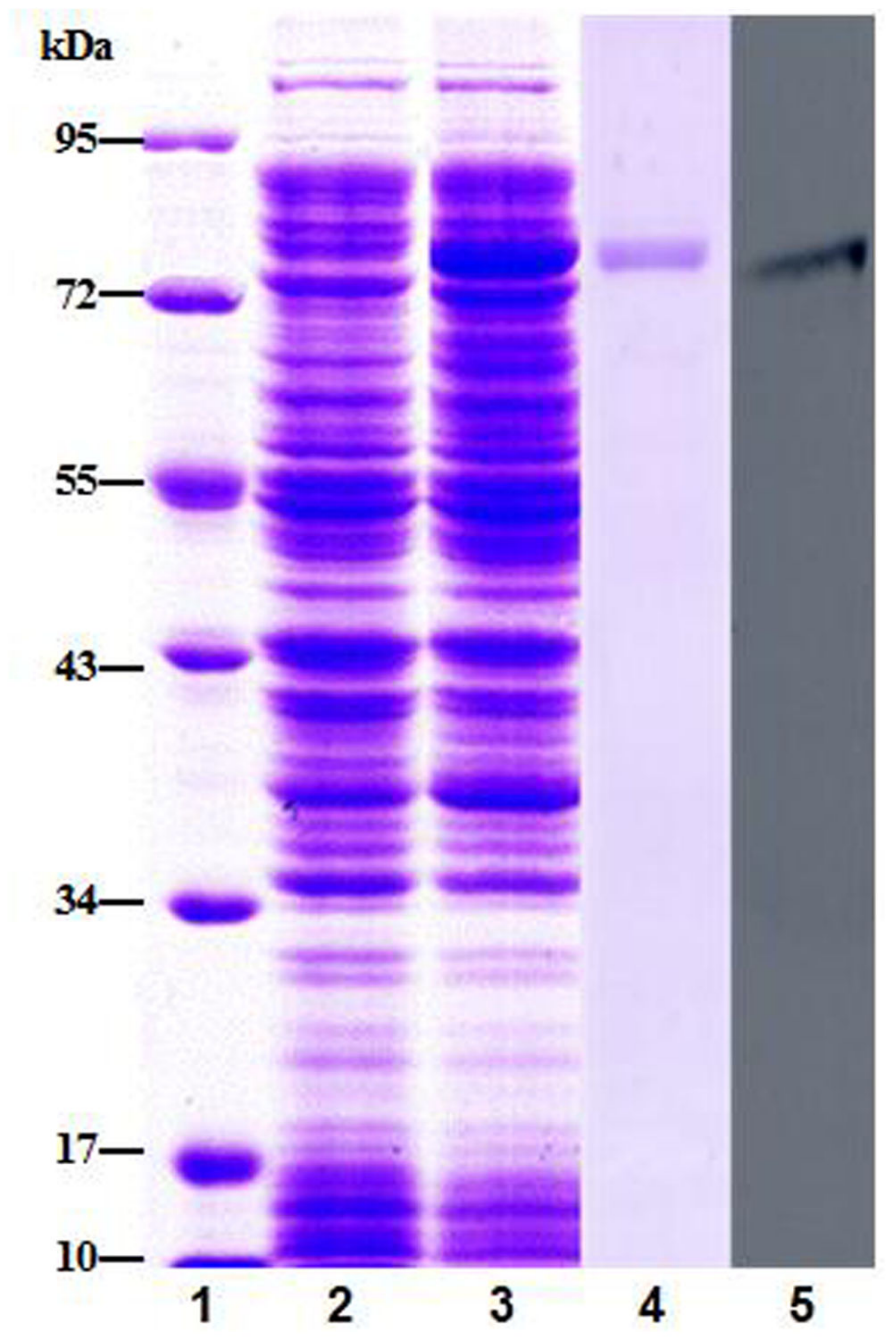
SDS-PAGE and western blotting analysis of rCfNOS. Lane 1: protein molecular standard; lane 2: negative control for rCfNOS (without induction); lane 3: induced rCfNOS; lane 4: purified rCfNOS; lane 5: western-blot of rCfNOS.

After IPTG induction, the whole cell lysate of *E. coli* BL21 (DE3)-transetta with *pEASY*-E1-CfNOS was analyzed by SDS-PAGE, and a distinct band with approximate molecular weight of 77 kDa was observed, which was consistent with the predicted molecular mass (Figure 3 in lane 3). There was no corresponding band in the cell lysate without induction (Figure 3 in lane 2). The purified rCfNOS of ~ 77 kDa (Figure 3 in lane 4) was used to immune the female Wistar rats to prepare the polyclonal antibody of rCfNOS. The specificity of the polyclonal antibody of rCfNOS was examined by western blotting. There was only a distinct band in the nitrocellulose membrane, whose molecular weight was in accordance with that of rCfNOS (Figure 3 in lane 5). Meanwhile, no distinct band was observed in the negative control.

### The activity of NOS

**Figure 4:**
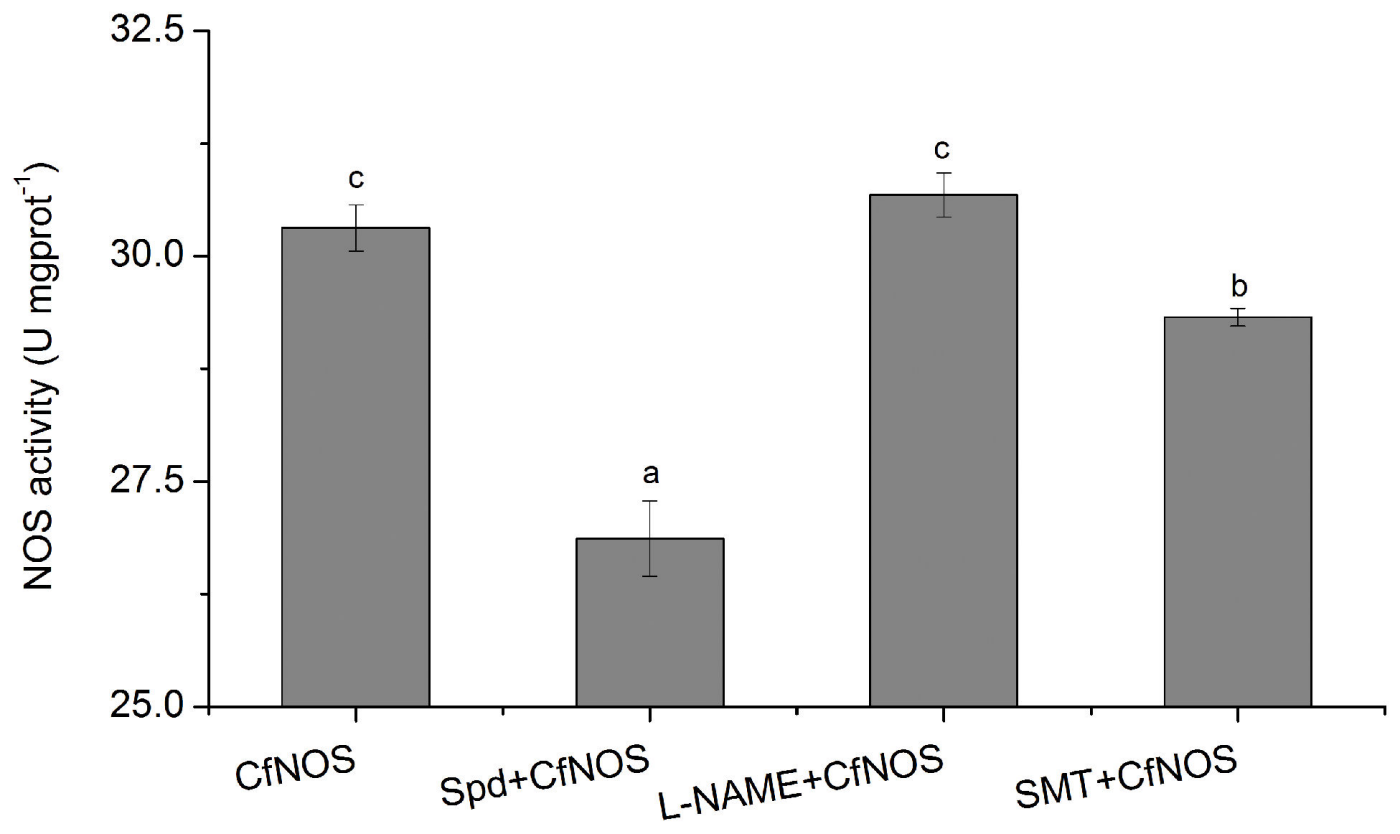
Detection of native CfNOS activity from haemocyte lysates. The protein of CfNOS was immunoprecipitated by polyclonal antibody of rCfNOS, and incubated with the elution buffer, Spd (0.01 mmol L^-1^), L-NAME (0.01 mmol L^-1^), and SMT (0.01 mmol L^-1^), respectively. CfNOS activity was detected by measuring the NO production using L-Arginine as the substrate. The elution buffer was used as blank. Results were expressed as unit activity per milligram protein (U mgprot^-1^). Each value was shown as mean ± SD (N = 3) and bars with different letters were significantly different (*P* < 0.05).

The native CfNOS protein was separated by immunoprecipitation and the activity was determined by NOS enzymatic assay. The activity of purified CfNOS protein was of 30.3 ± 0.3 U mgprot^-1^, whereas it decreased significantly to 26.9 ± 0.4 and 29.3 ± 0.1 U mgprot^-1^ (*P* < 0.01) after the addition of Spd and SMT, respectively (Figure 4). And its activity did not change significantly after the addition of L-NAME.

### Temporal expression of CfNOS mRNA during the early development of scallop larvae

**Figure 5:**
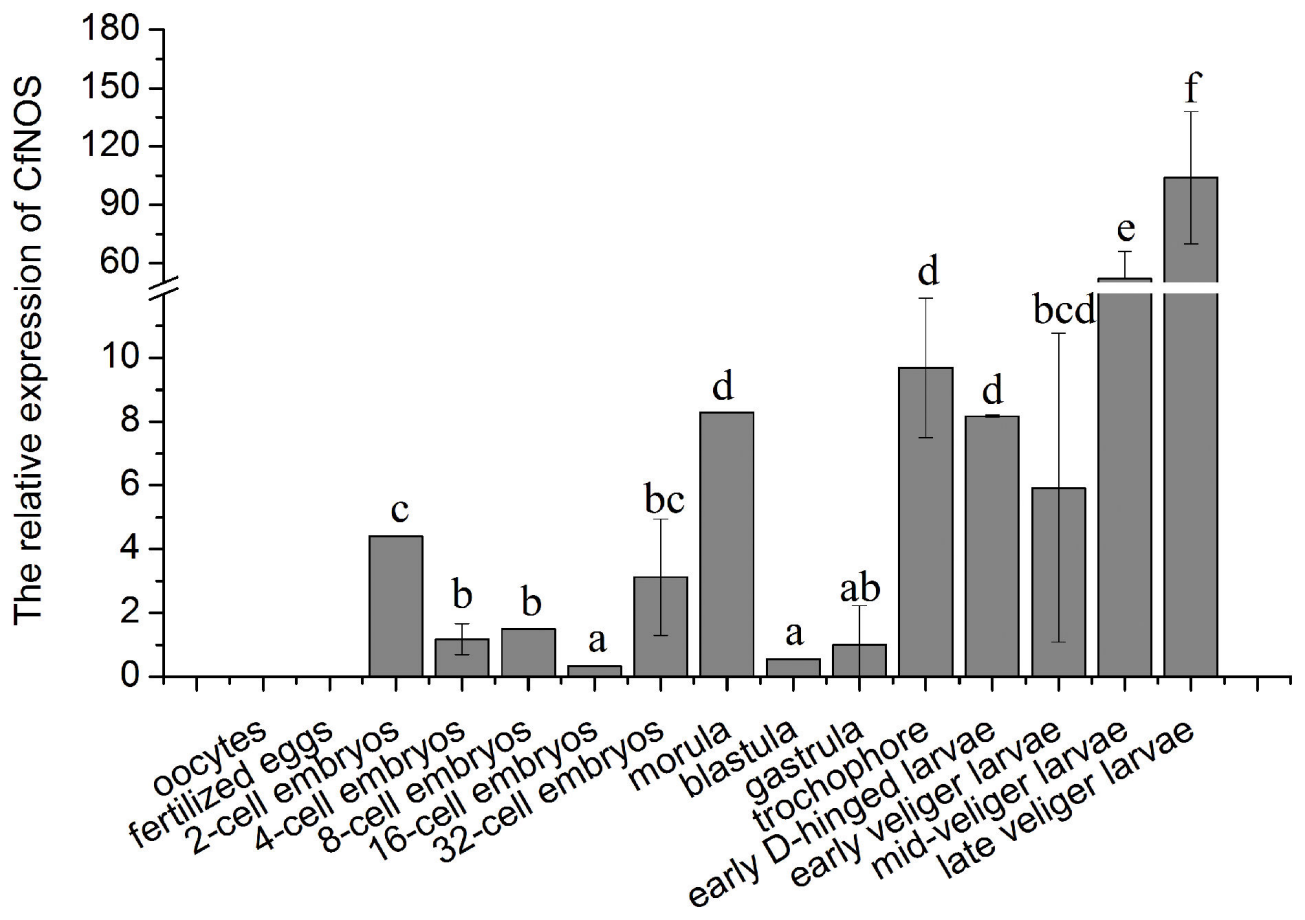
Temporal expression of CfNOS mRNA during ontogenesis in scallop *C* *. farreri* by quantitative real-time PCR. Elongation factor 1 alpha (EF-1a) gene was used as internal control to calibrate the cDNA template for all the samples. Each value was shown as mean ± SD (N = 6) and bars with different letters were significantly different (*P* < 0.05).

The quantitative real-time PCR was employed to investigate the temporal expression of CfNOS mRNA during the early development of scallop larvae. For CfNOS and CfEF-1α gene, there was only one peak at the corresponding melting temperature in the dissociation curve analysis, indicating that the PCR was specifically amplified (data not shown). The CfNOS transcripts could be detected in all embryos and larvae except for oocytes and fertilized eggs, and the expression level underwent three increases from 2-cell embryos to late veliger larvae referenced by that at gastrula stage (Figure 5). The first rising occurred at the 2-cell embryo stage (4.4-fold, *P* < 0.05), and the second one at the morula stage (8.3-fold, *P* < 0.05). The expression level of CfNOS mRNA started to increase thirdly at the gastrula stage, after which it was elevated dramatically to 52.1- and 104.0-fold (*P* < 0.01) at the mid- and late veliger larva stages, respectively.

### The distribution of CfNOS mRNA in different tissues

**Figure 6:**
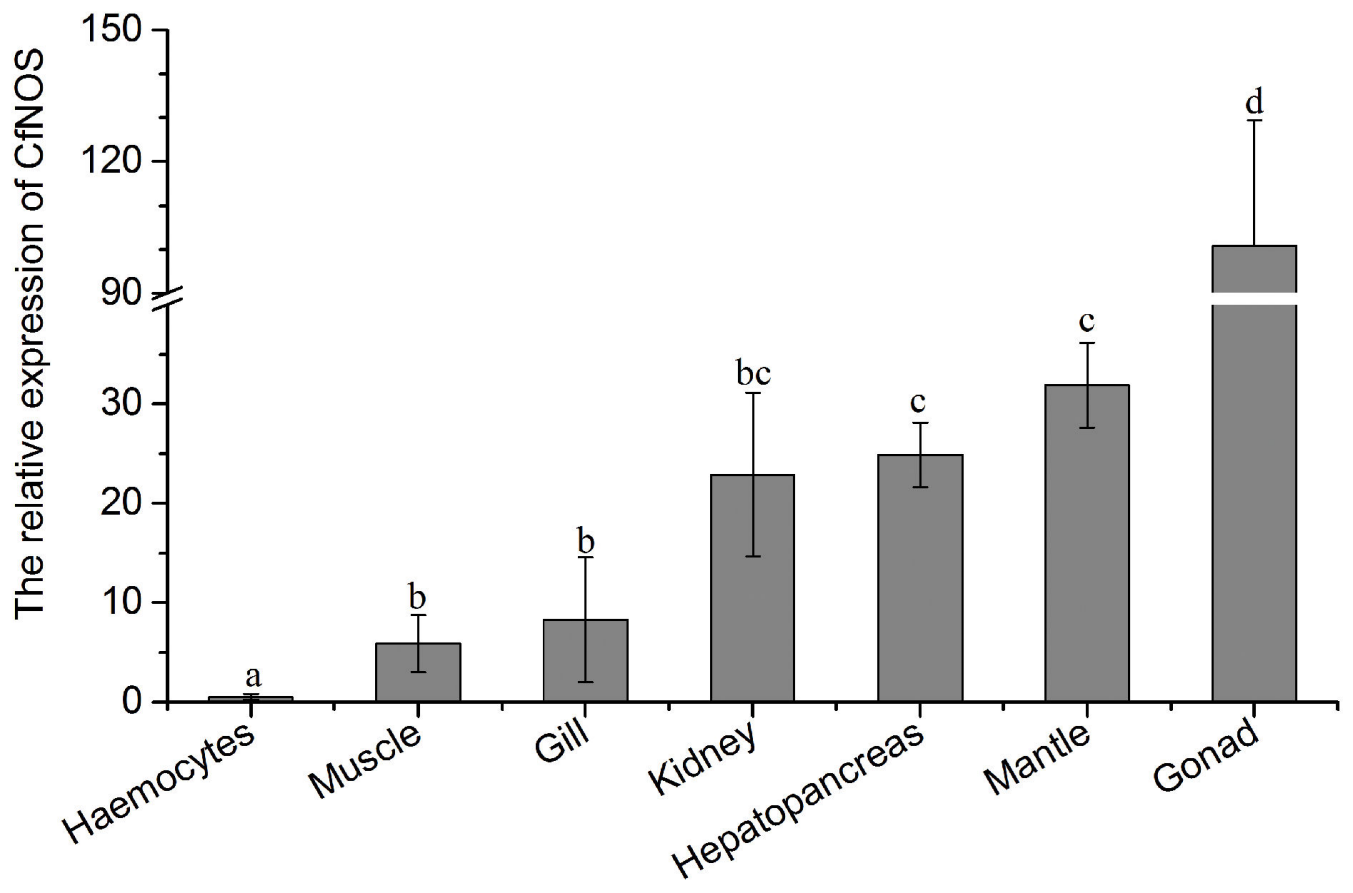
CfNOS mRNA expression levels in multiple scallop tissues detected by real-time PCR. CfNOS transcript levels in adductor muscle, gill, kidney, hepatopancreas, mantle and gonad are normalized to that of hemocytes. The gene of β-actin was used as internal control to calibrate the cDNA template for all the samples. Vertical bars represents the mean ± SD (N = 6) and bars with different letters were significantly different (*P* < 0.05).

The distribution of CfNOS mRNA in different tissue was investigated by quantitative real-time PCR with β-actin as internal control and the expression level of CfNOS mRNA in haemocytes served as reference. The CfNOS transcripts were ubiquitously detectable in all tested tissues, including haemocytes, muscle, gill, kidney, hepatopancreas, mantle and gonad. The highest expression level of CfNOS mRNA was found in gonad, which was 100.7-fold (*P* < 0.01) of that in haemocytes, following by the expression levels in mantle and hepatopancreas (31.9- and 24.9-fold, respectively, *P* < 0.01). The CfNOS mRNA expression levels in kidney (22.9-fold, *P* < 0.05), gill (8.3-fold, *P* < 0.05) and adductor muscle (5.9-fold, *P* < 0.05) were relatively lower, but still significantly higher than that in haemocytes (Figure 6).

### Localization of CfNOS protein in scallop haemocytes

**Figure 7:**
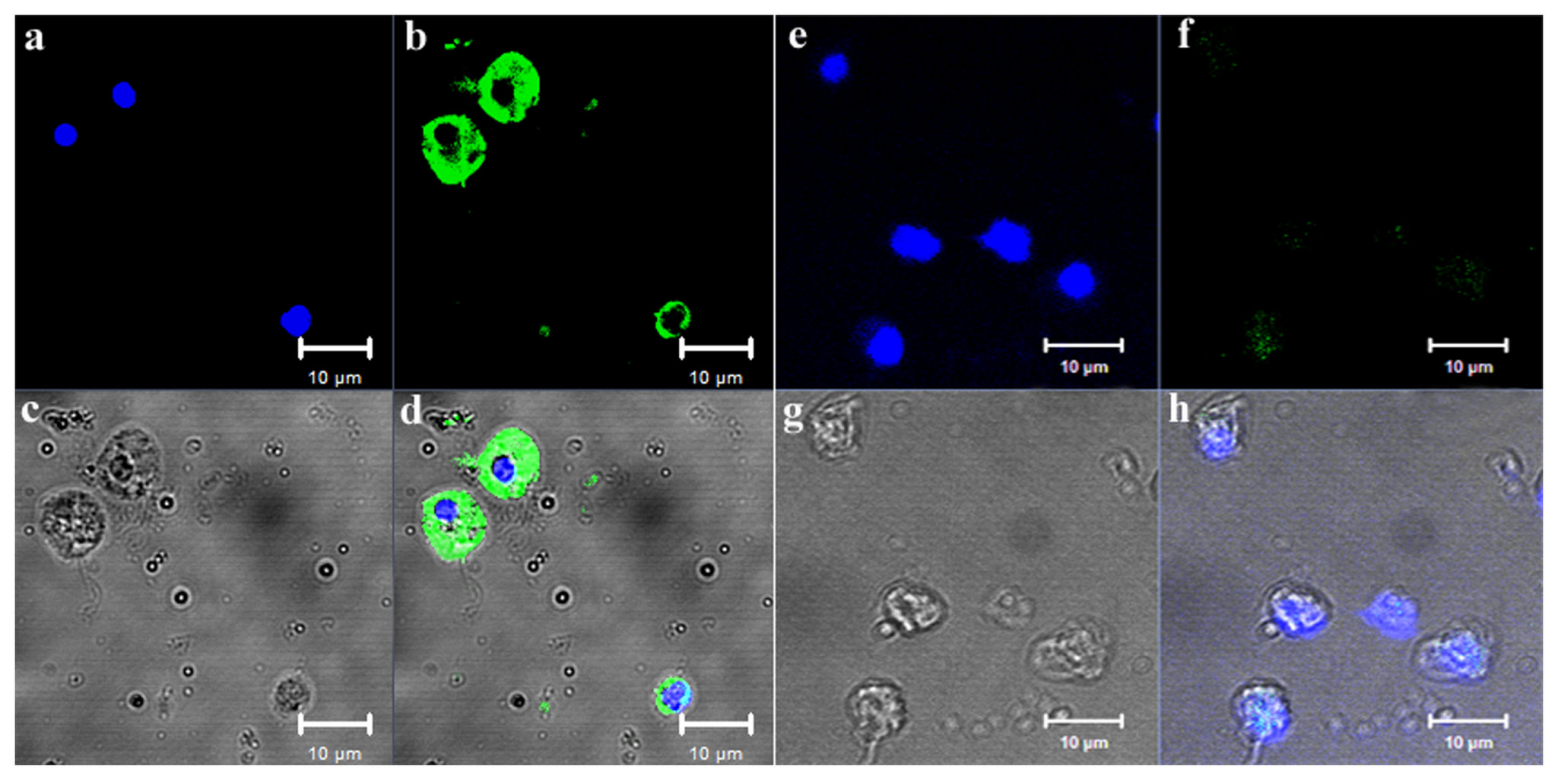
Localization of CfNOS protein in scallop haemocytes. Binding of antibody to CfNOS was visualized by Alexa 488-labeled secondary antibody (green), and the nucleus of haemocytes was stained with DAPI (blue). a–d: rat polyclonal antiserum to rCfNOS, bar = 10 µm; e-h: non-immune rat serum, bar = 10 µm.

In the scallop haemocytes, the nucleus stained by DAPI was observed in blue, and the CfNOS immunoreactive areas were in green (Figure 7). The positive signal of CfNOS appeared encompassing the nucleus and distributed over the cytoplasm and cell membrane (Figure 7a–d), while no positive signal was observed in the negative control (Figure 7e-f).

### The temporal expression of CfNOS mRNA after PAMPs stimulation

**Figure 8:**
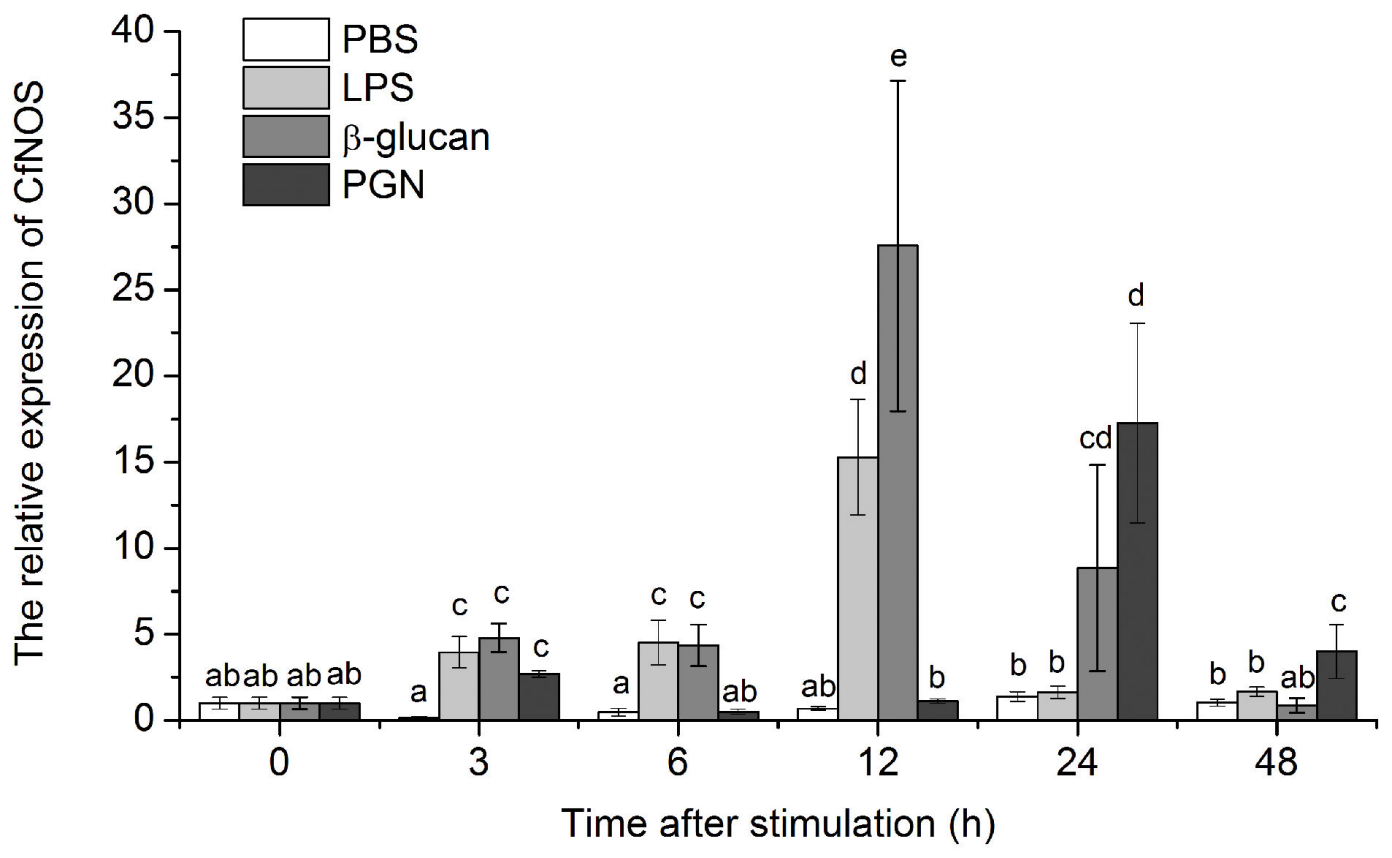
Temporal expression of CfNOS mRNA in scallop haemocytes after LPS, PGN, β-glucan and PBS challenge. The samples were collected after the treatments for 3, 6, 12, 24 and 48 h. Data was expressed as the ratio of the CfNOS mRNA to the β-actin mRNA. The scallops injected with PBS were used as the control group. The value was shown as mean ± SD (N = 6) and bars with different letters were significantly different (*P* < 0.05).

With the stimulation of PAMPs (LPS, β-glucan, PGN), the expression level of CfNOS mRNA in scallop haemocytes increased significantly, while there was no significant change in the control group treated by PBS (Figure 8).

After LPS stimulation, the CfNOS mRNA expression level started to increase at 3 h (4.0-fold, *P* < 0.01), reached the peak at 12 h (15.3-fold, *P* < 0.01), and decreased to control levels at 24 and 48 h. Similarly, it began to increase at 3 h (4.8-fold, *P* < 0.01) after β-glucan stimulation, following by a burst of increase to 27.6-fold (*P* < 0.01) at 12 h, subsequently decreased at 24 h (8.85-fold, *P* < 0.05), and recovered to the control level at 48 h. There were two increases observed during the PGN stimulated expression of CfNOS mRNA. The expression level increased at 3 h (2.7-fold, *P* < 0.01) for the first time, and peaked at 24 h (17.3-fold, *P* < 0.01) during the second rising.

### Temporal expression of CfNOS mRNA in haemocytes after TNF-α stimulation

**Figure 9:**
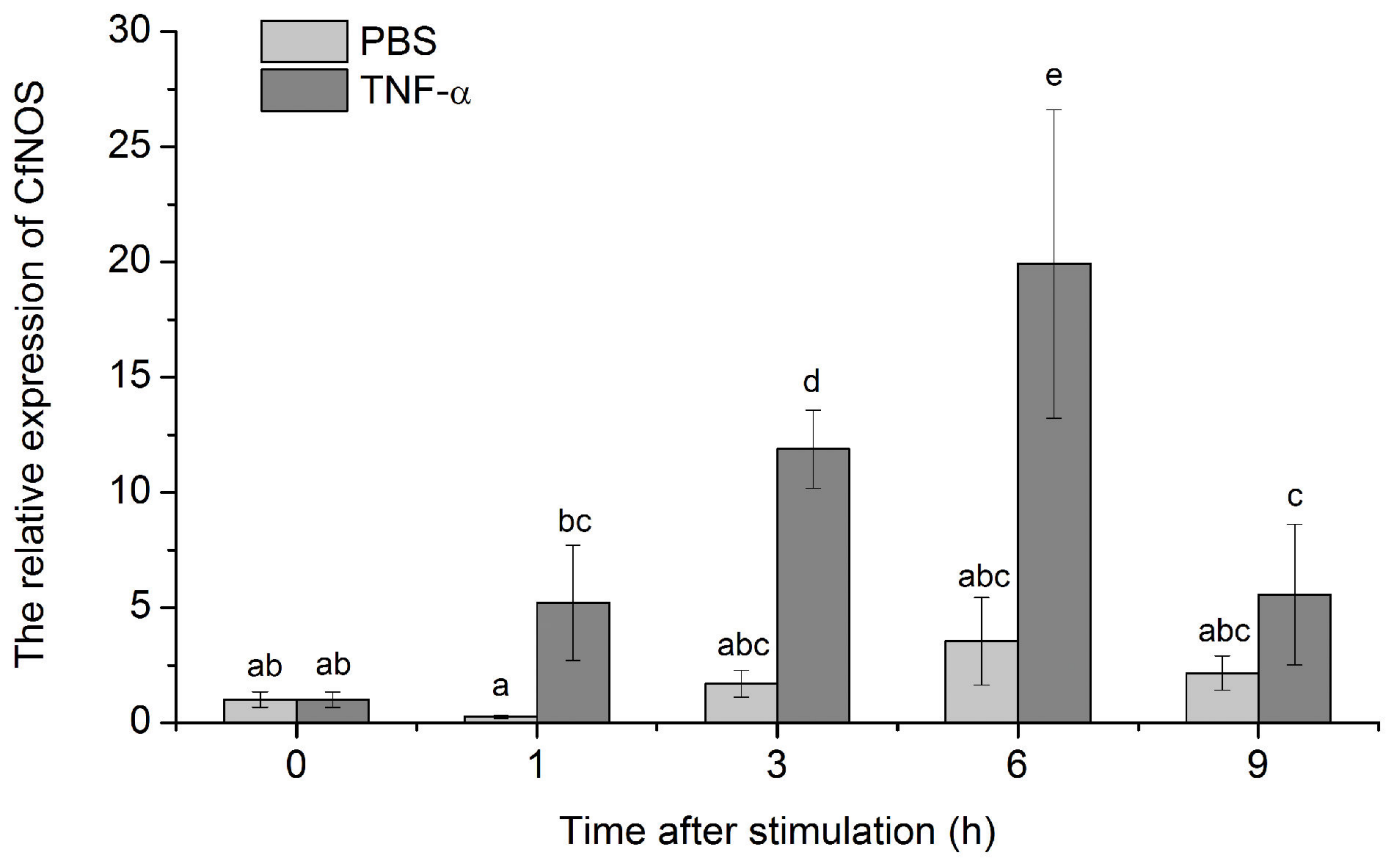
Temporal expression of CfNOS mRNA in scallop haemocytes after TNF-α stimulation. The samples were collected after the treatments for 1, 3, 6 and 9 h. Data was expressed as the ratio of the CfNOS mRNA to the β-actin mRNA. The scallops injected with PBS were used as the control group. Values were shown as mean ± SD (N = 6) and bars with different letters were significantly different (*P* < 0.05).

After the stimulation of TNF-α, the expression level of CfNOS mRNA in scallop haemocytes started to increase at 1 h (5.2-fold, *P* < 0.05), and peaked at 6 h (19.9-fold, *P* < 0.01), then declined to the same level with control group at 9 h (Figure 9). There was no significant change in the control group stimulated by PBS.

## Discussion

NOS is the most crucial member in NO system for its exclusive role in *de novo* synthesis of NO, and thus decisive in the physiological functions of NO system. It has beneficial microbicidal, antiviral, and antiparasital effects by producing high output of NO in both vertebrate and invertebrate. In the present study, an NOS gene was cloned from *C* *. farreri* (CfNOS) and its structure features were characterized. Its mRNA expressions were detected during scallop ontogenesis and in multiple tissues, and its localization in scallop haemocytes was observed. Moreover, the enzymatic activity of the native CfNOS protein and its immunological activity against PAMPs and TNF-α were examined for further understanding of scallop NOS family constitution and its role in immune defense.

The full-length cDNA of the CfNOS gene was of 5023 bp, containing a 5’ UTR of 140 bp, a 3’ UTR of 422 bp with a poly(A) tail, and an ORF of 4461 bp encoding a polypeptide of 1486 amino acids. The deduced amino acid sequence of CfNOS shared 38.8~45.3, 50~54.7, 42.5~44.5 and 40.7~47.0% similarities with arthropod NOSs, chordate nNOSs, iNOSs, and eNOSs, respectively. SMART program and multiple sequence alignments revealed that CfNOS contained PDZ, oxygenase, and reductase domains. There was an additional autoinhibitory loop in the FMN binding region of CfNOS. The presence of both PDZ domain and autoinhibitory loop indicated that the structure of CfNOS was similar to that of nNOS. In ML phylogenic tree, the CfNOS was clustered into the mollusc NOS group, and then gathered together with the groups of arthropod NOSs and deuterostome NOSs, which contained nNOSs, eNOSs and iNOSs. This evolution relationship implied that the three-isoform constitution of NOS family might be absent in invertebrate, and there might exist only one ancestral NOS, that is, only one NOS isoform in mollusc [38]. Besides, the enzymatic activity of isolated CfNOS protein from haemocytes was determined to be 30.3 ± 0.3 U mgprot^-1^. The molecular characters, sequence similarity and the catalytic activity comprehensively suggested that CfNOS was the homologue of NOS in scallop *C* *. farreri* and structurally similar to deuterostome nNOS, and furthermore, the phylogenetic relationship indicated that CfNOS might be the exclusive isoform in scallop NOS family.

NO system has been reported to regulate the metabolism, growth, cell proliferation, development, and neurogenesis in vertebrates, insects and molluscs [39–41], among which the expressions of NOSs endow NO with versatile roles in microvascular, neural and immune systems [11,42,43]. In order to investigate the potential roles of scallop NO system, the expressions of CfNOS mRNA during ontogenesis and in different tissues were examined in the present study.

The temporal expression of CfNOS mRNA was determined in scallop embryos and larvae stages to understand the development of NO system in the early development of scallop. The CfNOS mRNA transcripts in oocytes and fertilized eggs were undetectable, possibly due to their absence in gametes or the dilemma that maternal transcripts were degraded completely and the novel transcription process had not been initiated [44]. The CfNOS mRNA transcripts were expressed from 2-cell embryos to late veliger larvae stages, and were observed in extremely dramatical levels at the mid- and late veliger larva stages. The present increase of CfNOS transcripts in trochophore might be attributable to the involvement of NO in cilia-driven rotational behavior [45]. The remarkably significant increases in mid- and late veliger stages indicated that these two stages were important periods for the development of NO system in scallop larvae, and in turn, implied that NO system might play an important part in the developmental stages.

The mRNA transcripts of CfNOS were also detected in various scallop tissues, including haemocytes, hepatopancreas, kidney, muscle, mantle, gill and gonad. The CfNOS transcripts were detectable in all the tested tissues, suggesting that CfNOS might be involved in versatile physiological processes [17]. The highest expression level of CfNOS mRNA was observed in the gonad, indicating that NO might be able to regulate scallop gametogenesis and/or steroidogenesis [16,18]. Relative higher level of CfNOS transcripts was expressed in the mantle, which suggested the possible role of NO system in sensory process [21]. In addition, as hepatopancreas and haemocytes are most important immune tissues in mollusc, the CfNOS transcripts in these tissues indicated that CfNOS might be involved in scallop immune responses [19,20]. Furthermore, the CfNOS protein was observed in the cytoplasm and cell membrane of scallop haemocytes. The presence of CfNOS mRNA transcripts and protein in scallop haemocytes suggested the possible involvement of CfNOS in scallop haemocyte immunity.

In order to examine the immunological role of CfNOS, the temporal expression of CfNOS mRNA in scallop haemocytes was detected after PAMPs stimulation. The transcripts of CfNOS mRNA started to express significantly at 3 h and the expression level peaked at 12 h after LPS and β-glucan stimulation, while increased significantly the second time at 24 h after PGN stimulation. The results revealed that the expression of CfNOS mRNA could be quickly triggered by different immune stimuli, suggesting the inducible activity of CfNOS during scallop immune defense and the involvement of CfNOS in a broad-spectrum of immune responses against the fungi, Gram-negative and -positive bacteria. Mollusc NO has been reported to be involved in the immune defense [46–48], and the iNOS-like activity was detected after the stimulation of LPS in the haemocytes of scallop *C* *. farreri* [49]. In vertebrate, iNOS can be induced by immune stimuli to synthesize high amounts of NO and is the main isoform in NOS family to function in immune defense. Comprehensively, the present results demonstrated that the mRNA expression and activity of CfNOS were inducible during the immune defense, and it might act homologous role of vertebrate iNOS in scallop.

The response of CfNOS transcripts in scallop haemocytes against TNF-α stimulation was also observed, so as to further investigate the activation mechanism of CfNOS during the immune responses. Significant increases of CfNOS mRNA expression firstly appeared at 1 h after the treatment with 50.0 ng mL^-1^ TNF-α, and then reached the peak at 6 h, revealing that the expression of CfNOS mRNA could be induced by TNF-α fleetly. In mammals, TNF activity was a useful monitor of LPS effects and it could cause a range of immune responses similar to those evoked by LPS itself, including the responses of NO, O_2_^-^, and other immune molecules [50]. Recently, TNF-α homologues have been identified in some invertebrates [51,52] and they were also validated to be involved in the responses against LPS [53], suggesting that invertebrate TNF-α might also act as the mediator of LPS-induced immune defense. Combined with the present results that the expression of CfNOS mRNA induced by LPS was delayed or at a relatively lower level compared with that induced by TNF-α, it was implied that the induction effect of LPS on the CfNOS expression might be brought about, at least in part, by its stimulatory effects on the formation of TNF-α.

In the immunoprecipitation and activity assay, CfNOS activity significantly decreased after the treatment of nNOS and iNOS inhibitors, while no significant change was observed after the treatment with inhibition of eNOS. It suggested that CfNOS possessed similar characterization of biochemical activity to nNOS and iNOS from vertebrates, and it might play analogously physiological roles in scallop. Thus, the responses of CfNOS against PAMPs and TNF-α treatment implied that it took the immunological role of iNOS to conduct the immunomodulation in scallop haemocytes in previous study [49]. The synthesized NO can react with other oxygen species to form highly toxic radicals, such as peroxynitrite (OONO^-^), to remove the external intrusions [49].

In conclusion, the present study cloned and characterized a novel gene of NOS from scallop *C* *. farreri* (CfNOS). It was structurally similar to nNOS and might be the only member in scallop NOS family. However, it got involved in the immune defense of scallop haemocytes and played the immunological role of iNOS, and its inducible activity against LPS stimulation might be preceded by the induction of TNF-α.
